# The rate of success of the conservative management of liver trauma in a developing country

**DOI:** 10.1186/s13017-017-0135-4

**Published:** 2017-06-07

**Authors:** S. Buci, M. Torba, A. Gjata, I. Kajo, Gj. Bushi, K. Kagjini

**Affiliations:** 1 Service of General Surgery, Trauma University Hospital, Tirana, Albania; 2 Department of Surgery, UHC “Mother Teresa”, Tirana, Albania; 3 Department of Internal Medicine, Trauma University Hospital, Tirana, Albania

**Keywords:** Liver trauma, Grade of injuries, Conservative treatment, Success

## Abstract

**Background:**

The conservative treatment of liver trauma has made important progress over the last 10 years at the Trauma University Hospital in Tirana, Albania. The percentage of success was 58.7%. The aims of this study were to analyze the conservative treatment of liver trauma and to compare the results with those in the literature.

**Methods:**

This study was conducted prospectively from January 2009 to December 2012. We analyzed 173 patients admitted to our hospital with liver trauma. Liver injuries were evaluated according to the American Association for the Surgery of Trauma and the World Society of Emergency Surgery classification, while the anatomic gravity of the associated injuries was defined using the Injury Severity Score system. The potential mortality was estimated with the Revised Trauma Score.

**Results:**

Out of the 173 patients with liver trauma, 83.2% were male. The main cause of liver trauma was motor vehicle crashes (50.9%). Blunt trauma was the cause of liver injury in 129 cases (74.6%), and penetrating trauma occurred in 44 cases (25.4%). Initially, the decision was to manage 88 cases (50.9%) via the conservative approach. Of these, 73 cases (42.2%) were successfully treated with conservative treatment, while in 15 cases (17.2%), this approach failed. The success rate of conservative treatment by grade of injuries was as follows: grade I (38.4%), grade II (30.1%), grade III (28.8%), and grade IV (2.7%). The likelihood of the success of conservative treatment had a significant correlation with the grade of the liver injury (*p* < 0.00001), associated intra-abdominal injuries (*p* = 0.00051), and complications (*z* = 2.3169, *p* = 0.02051). The overall mortality rate of liver trauma was 13.2%.

**Conclusions:**

The likelihood of success in using conservative treatment had a significant correlation with the grade of liver injury and associated intra-abdominal injuries. The limited hospital resources and low level of consensus on conservative treatment had a negative impact on the level of success.

## Background

Currently, the conservative management of liver trauma is considered the “Gold Standard.” More than 80% of patients with non-severe liver injuries are treated successfully without surgery [1–4]. Nance and Cohn support the use of conservative treatment in hemodynamically stable patients who do not exhibit signs of peritoneal irritation [5]. In Albania, the conservative treatment of liver trauma has made important progress over the last decade. The diagnostic and therapeutic strategy for liver injury depends on the hemodynamic status of the traumatized patients and the associated injuries.

The World Society of Emergency Surgery (WSES) liver trauma classification considers not only the anatomic American Association for the Surgery of Trauma (AAST) classification but also, more importantly, the hemodynamic status and the associated injuries [6] (Table 1).

**Table 1:** WSES liver trauma classification

	WSES grade	Blunt/penetrating (stab/guns)	AAST	Hemodynamic	CT scan	First-line treatment
Minor	WSES grade I	B/P	I–II	Stable		
		SW/GSW				
Moderate	WSES grade II	B/P	III	Stable	Yes	NOM*
		SW/GSW			Local Exploration in SW	Serial Clinical/Laboratory/Radiological Evaluation
Severe	WSES grade III	B/P	IV–V	Stable		
		SW/GSW				
	WSES grade IV	B/P	I–VI	Unstable	No	OM
		SW/GSW				

*SW* stab wound, *GSW* gunshot wound, *OM* operative management, * *NOM* non-operative management

The abdominal echography procedure is essential in the emergency department for patients with hemodynamic instability. Currently, computerized tomography is the preferred method to evaluate blunt liver trauma in hemodynamically stable patients or in patients who have been stabilized after an initial fluid resuscitation [7].

The conservative treatment of liver injury can be initiated when there is a possibility of monitoring the traumatized patient clinically, biologically, and radiologically in the correct manner and when there is a close collaboration between surgeons, intensivists, and radiologists. The failure of conservative treatment occurs in 15% of cases, often in patients with extrahepatic injuries or white laparotomy [8, 9].

The objectives of this study were to analyze the conservative management of liver trauma, the likelihood for success, and the causes of failure and to compare the results with those in the literature.

## Methods

This is a prospective study performed from January 2009 to December 2012. We analyzed 173 patients with hepatobiliary trauma who were admitted to the Trauma University Hospital, Tirana, Albania. Liver injuries were evaluated according to AAST and WSES classifications via ultrasonography and CT scan. The anatomic gravity of the associated injuries was defined using the Injury Severity Score (ISS) system. The potential mortality was estimated using the Revised Trauma Score (RTS). A simple ordinary least square regression (OLS) of several factors on the success of conservative treatment of trauma injury patient reveals the results of the following table. For this analysis, the variables are Surviv (whether the patient survives the treatment of the injury [No = 0; Yes = 1]); Age (age of the patient [6–15 years old = 1; 16–25 years old = 2; 26–35 years old = 3; 36–45 years old = 4; 46–55 years old = 5; 56–65 years old = 6; 66–75 years old = 7]); Gender (gender [Male = 1; Female = 0]); Cinjur (if only the liver is injured, then the value is 0. For all other combinations, like the liver and head and the liver and thorax, the variable takes the value of 1); Grade (grade [the six-grade scale standard is used: Minor = 1; Moderate = 2; Serious = 3; Severe = 4; Critical = 5; Un-survivable = 6]); Conserv (conservative treatment of the patient [Yes = 1; No = 0]); Interv (the patient is treated with an intervention [Yes = 1; No = 0]); ISS (anatomical; the higher the number, the more severe the injury); and RTS (psychological; the lower the number, the more severe the condition of the patient).

Treatment for each patient was chosen based on the set of indicators for laparotomy and under the consideration for conservative treatment. The indicators were (1) hemodynamic stability, (2) presence/absence of peritoneal irritation signs, (3) identification and evaluation of CT scan grade of liver injuries, (4) hemoperitoneum <500 ml, and (5) the absence of injury to cavitare organs [10, 11], based on the algorithm for the non-operative management of blunt hepatic trauma [12].

Based on these criteria, patients were divided into two main groups: group A (including 88 patients who met the conditions for conservative treatment) and group B (including 85 patients who had indications for immediate laparotomy).

## Results

The average age of patients with hepatic trauma was 23.4 years old (ranging from 6 to 75), including 83.2% male and 16.8% female patients. 

In our study, blunt trauma was the cause of liver injury in 129 cases (74.6%), while penetrating trauma occurred in 44 cases (25.4%).

The causes of hepatic trauma were motor vehicle crashes in 88 cases (50.9%), falls from height in 32 cases (18.4%), gunshot wounds in 24 cases (13.8%), sharp tools in 19 cases (11%), direct blows in 8 cases (4.7%), iatrogenic in 1 case (0.6%), and hepatic trauma from an electrical arc in 1 case (0.6%) (Tables 2 and 3).

**Table 2:** Hemodynamic status

Initial hemodynamic status	No. of patients	Percentage
Hemodynamic instability	47	27.1
Stabilized after intravenous liquid administration	84	48.6
Hemodynamic stability	42	24.3

**Table 3:** Hematocrit level (at the time of arrival in the ER)

Initial hematocrit level	No. of patients	Percentage
Up to 37	124	71.7
37-30	34	19.7
Under 30	15	8.6

Alanine aminotransferase (ALT) >100 U/l and aspartate aminotransferase (AST) >200 U/l were found in 96 patients (55.5%), and ALT >1000 U/l and AST >1100 U/l were found in 2 patients (1.6%).

Diagnostic peritoneal lavage (DPL) was used in10 patients (5.6%). In 6 patients, we found intestine leak.

In our study, 62 patients (35.9%) were transfused with 1 unit of blood, 41 (23.8%) were transfused with 2 units, 30 (17.9%) were transfused with 3 units, 22 (12.8%) were transfused with 4 units, and 17 (9.6%) were transfused with more than 4 units (Table 4).

**Table 4:** Hepatic injuries according to AAST grade

Grade	Number of cases	Percentage
I	36	20.8
II	60	34.7
III	47	27.2
IV	21	12.1
V	8	4.6
VI	1	0.6

The injury frequencies, according to the Couinaud segment, were as follows: I segment (*n* = 8) 2.5%, II segment (*n* = 10) 3.1%, III segment (*n* = 16) 5%, IV segment (*n* = 32) 10%, V segment (*n* = 49) 15.3%, VI segment (*n* = 76) 23.8%, VII segment (*n* = 65) 20.3%, and VIII segment (*n* = 64) 20%.

The frequency of liver injury according to the WSES were WSES grade I (*n* = 65) 37.6%, WSES grade II (*n* = 55) 31.8%, WSES grade III (*n* = 6) 3.5%, and WSES grade IV (*n* = 47) 27.1%.

Isolated hepatic injuries were detected in 45 cases (26.1%), and combined hepatic injuries were present in 128 cases (73.9%).

Hepatic injuries were associated with intra-abdominal hollow organ injuries in 33 cases (19.1%) and with parenchymal injuries in 31 cases (17.9%) (Table 5).

**Table 5:** The mechanism of trauma and associated abdominal injuries

Organ	Blunt trauma	Penetrating trauma	Total	Percentage
Spleen	14	2	16	9.2
Kidneys	9	3	12	6.9
Pancreas	2	2	4	2.3
Stomach	3	7	10	5.8
Small intestine *+* duodenum	3	9	12	6.9
Large intestine + rectum	3	6	9	5.2
Diaphragm	8	27	35	20.2
Esophagus	0	1	1	0.6
Urinary bladder	1	0	2	1.2
Total	43	57	100	58.3

Hepatic injuries were associated with extra-abdominal injuries, including the head in 27 cases (15.6%), the thorax and diaphragm in 80 cases (46.2%), the heart in 2 cases (1.2%), the vertebral column in 3 cases (1.7%), pelvic fracture in 12 cases (6.9%), and limb fractures in 19 cases (11%) (Tables 6 and 7).

**Table 6:** Success of conservative management according to AAST grade

Grade	Number of cases	Percentage
I	28	38.4
II	22	30.1
III	21	28.8
IV	2	2.7
V	0	0
VI	0	0

**Table 7:** Management according the WSES grade

WSES	Conservative	Laparotomy
WSES grade I	50 (76.9%)	15 (23.1%)
WSES grade II	21 (38.2%)	34 (61.8%)
WSES grade III	2 (33.3%)	4 (66.7%)
WSES grade IV	0 (0%)	47 (100%)

In group A (conservative treatment of 88 patients), 73 patients (42.2%) benefitted from successful conservative treatment. Unsuccessful conservative treatment due to further complications was seen in 15 patients (17.2%). In patients with isolated liver injuries, conservative treatment was successful in 27 cases (58.7%).

In group B, 100 patients underwent laparotomy. An immediate laparotomy was performed in 85 patients (49.1%), a laparotomy for hepatic injury was performed in 30 patients (30%), laparotomy for extrahepatic injury was performed in 70 patients (70%), perihepatic packing was performed in 10 patients (11.7%), and relaparotomy was performed in 6 patients (7%). Extrahepatic injuries included the stomach (*n* = 10, 12.3%), the small intestine and duodenum (*n* = 12, 14.8%), the large intestine and rectum (*n* = 9, 11.1%), the spleen (*n* = 16, 19.7%), the kidneys (*n* = 12, 14.8%), the pancreas (*n* = 4, 4.9%), the diaphragm (*n* = 35, 43.2%), the intra-abdominal esophagus (*n* = 1, 1.2%), the cholecyst (*n* = 3, 3.7%), and the urinary bladder (*n* = 2, 2.5%).

We observed that 10.9% of the patients who were treated in a conservative manner had an ISS of ≥20. We also observed that in this group of patients, 86.3% had an RTS of >5 (Tables 8, 9, 10, and 11).

**Table 8:** The causes of failure of conservative treatment

Complications	No. of patients	Percentage
Secondary hemorrhage	3	3.4
Biliary peritonitis	2	2.3
Intrahepatic biloma	1	1.1
Extrahepatic biloma	2	2.3
Hollow organ injuries	2	2.3
Liver compartment syndrome	1	1.1
Gangrenous cholecystitis	2	2.3
Peritoneal inflammatory syndrome	2	2.3
Total	15	17.2

**Table 9:** Complications of conservative treatment of patients with combined liver trauma according to some authors

Complications	References	Our study (%)
Secondary hemorrhage	5% [30]	3.4
Missed injuries	3–5% [28, 35]	2.3
Hemobilia	0.3–1.2% [36, 37]	1
Bilhemia	0.2–1% [38]	1
Bile leakage	3–20%	6.8

**Table 10:** Comparative data on secondary indications for surgical treatment after an initial determination to perform conservative treatment

Secondary indications for surgical treatment	Letoublon et al. (186 patients) (%)	Our study (173 patients) (%)
Peritoneal inflammatory syndrome	5.5	2.3
Secondary hemorrhage	1	3.4
Liver compartment syndrome	1	1.1
Hollow organ injuries	1	2.3
Biliary peritonitis	0.5	2.3
Extrahepatic biloma	0	2.3
Post-traumatic cholecystitis	0	2.3
Intrahepatic biloma	0	1.1
Bilothorax	0.5	0
Abdominal compartment syndrome	1	0
Post-traumatic pancreatitis	0.5	0

**Table 11:** Frequencies of several variables for both treatment modes (conservative and laparotomy)

Variable	Conservative	Laparotomy
Male	61 (42.4%)	83 (57.6%)
Female	12 (41.4%)	17 (58.6%)
>55 years old	8 (57.1%)	6 (42.9%)
<55 years old	65 (40.9%)	94 (59.1%)
ISS >20	8 (8.8)	83 (91.2%)
ISS <20	65 (79.3%)	17 (20.7%)
RTS >5	63 (63.6%)	36 (36.4%)
RTS <5	10 (13.5%)	64 (86.5%)
≤Grade IV	73 (42.1%)	100 (57.9%)
>Grade IV	0 (0%)	9 (100%)
Complications	15 (20.5%)	11 (11%)
Mortality	1 (1.4%)	12 (12%)

The chances of successful conservative treatment significantly depend on the grade of liver injury (*p* < 0.00001). There is also a statistically significant connection between the successful conservative treatment of liver trauma and a combination of injuries with other organs (*p* value = 0.00051) and complications (*z* = 2.3169, *p* = 0.02051) (Table 12).

**Table 12:** OLS, “success of the conservative management” as the dependent variable

Variables	Coefficient	Std. error	*t* ratio	*p* value
Const	−0.233669	0.311065	−0.7512	0.4536
Age	0.0493656	0.0196583	2.5112	0.0130
Gender	0.0253333	0.0731743	0.3462	0.7296
Cinjur	0.136536	0.0745459	1.8316	0.0688
Grade	0.0414623	0.0336495	1.2322	0.2196
Resec	−0.151871	0.134283	−1.1310	0.2597
ISS	−0.0124758	0.0027189	−4.5885	<0.0001
RTS	0.0892609	0.0315478	2.8294	0.0052
Mean dependent var.	0.445087	S.D. dependent var.		0.498418
Sum-squared resid.	27.91268	S.E. of regression		0.411300
*R*-squared	0.346740	Adjusted *R*-squared		0.319026

The OLS shows that the patient age, gender, combined injury, and grade of injury (anatomical, physiological, and locational) account for more than 30% of the variation in the success of conservative treatment (as seen with an adjusted *R*-squared = 0.319). From the variables under consideration, it is clear that patient age, combination of injuries with other organs besides the liver, grade of injury, and RTS (psychological trauma) are statistically important. This is verifiable from the above table that presents both the *p* values and the *t* values.

The overall mortality rate of liver trauma was 13.2%.

## Discussion

Our study demonstrated that liver injuries occurred in 17% of patients with abdominal trauma. Shanmuganathan et al. have reported that liver injuries occurred in 20% of patients with blunt abdominal trauma [13]. We found that male to female ratio was 5:1. Beel et al. found that the male to female ratio varies from 15:1 [14]. Approximately 15–20 years ago, all traumatic liver injuries were treated surgically, but in 50–80% of cases, no active bleeding was found [15, 16]. We also found liver injuries with no active bleeding during laparotomy for associated injuries.

In our study, the hemodynamic status was the main criterion in determining the therapeutic approach. Approximately 85% of patients with blunt liver trauma are hemodynamically stable or stabilize after receiving intravenous liquids [17], which corresponds with the findings in our study. Richardson et al. commented that many experienced surgeons in trauma surgery apply surgical treatment in hemodynamically stable patients and they have concluded that conservative treatment has a positive impact on patient survival [18]. In hemodynamically stable patients, a helical CT examination with oral and venous contrast was performed to determine the grade of the liver injury, the amount of hemoperitoneum, the presence of pseudoaneurysms, and the presence of other intraperitoneal injuries.

Hemoperitoneum was observed in repeated ultrasound examinations, and in some cases, re-evaluation was done via CT scan. Malhotra et al. reports that a large amount of hemoperitoneum (when blood is present in the lateral channels, the perihepatic space, and the Douglas pouch) is a significant risk factor for the failure of conservative treatment [19]. Due to limited resources for transfusion in our hospital, we interrupted conservative treatment in patients who had a considerable need for transfusion and when the amount of hemoperitoneum was determined to be progressively increasing.

From group A of 88 patients selected for conservative management, 15 patients showed complications that necessitated surgery. Group B consisted of 85 patients who underwent immediate laporatomy due to the following indications: hemodynamic instability, associated intra-abdominal injuries, and penetrating trauma. It is worth emphasizing that a case with penetrating liver trauma caused by hunting rifle wounds was managed conservatively. Ten patients underwent perihepatic packing; of these, a second laparotomy was performed in 6 patients and 4 patients did not survive.

In our study, we found that gunshot wounds and wounds caused by sharp tools had an incidence of 24.8%.

This percentage was significant and contributed to a reduction in the number of patients managed conservatively. The gunshot wounds were penetrating in 35–70% of cases [20], and the wounds caused by sharp tools were not penetrating in 35–61% of cases [21, 22].

Diagnostic peritoneal lavage (DPL) is another valid tool to determine the presence or nature of intraperitoneal liquids. We used this procedure in a few cases. DPL, described by Root in 1965, remains an important tool in the hands of surgeons, especially in the absence of minimally invasive equipment. DPL has a very high sensibility and specificity rate for intraperitoneal injuries, 95 and 99%, respectively. However, this procedure is associated with complications in 0.8–1.7% of cases [23].

Our study showed that conservative treatment turned out to be successful in 42.2% of patients with combined hepatic trauma and in 58.7% of patients with isolated hepatic trauma. These percentages recorded in our study are lower than those reported elsewhere. It is our view that these differences are related to two factors: (1) a low level of consensus for conservative management and (2) limited hospital resources (limited interventional radiology procedures). Conservative treatment failed in 17.2% of cases. In some studies, it has been reported that the efficacy of conservative management of liver trauma in hemodynamically stable patients is between 87 and 98%, and the failure rate is between 10 and 25% [24, 25].

Injuries that were grade III or worse added substantially to the failure of conservative management. We had only two patients with grade IV liver injury that were successfully managed conservatively. Malhotra et al. found that in 14% of patients with grade IV injuries and 22.6% of patients with grade V injuries, conservative treatment was not successful, and the failure rate in patients with grades I, II, and III injuries was 3–7 and 5% [19]. The likelihood of the success of conservative treatment had a significant correlation with the grade of liver injury. The coefficient of grade injury was −2.6, which means that for every increase in the grade of the injury, the likelihood of the success of conservative treatment drops to 2.6. In addition, the success of conservative treatment had a significant correlation with associated intra-abdominal injuries. Other factors were statistically insignificant in the success of the conservative treatment.

The failure of the conservative treatment was often attributed to the deterioration of hemodynamic parameters, bile leakage, and the presence of overlapping septic complications. Durham et al. and Hammond et al. have reported that secondary hemorrhage occurs in less than 5% of cases treated conservatively [26, 27]. We observed that the failure of conservative treatment due to secondary hemorrhage occurred in 3% of cases. Buckman et al. showed that bile leakage can occur in 3–20% of patients who are managed conservatively [28, 29]. The frequency of hemobilia, as reported by Croce et al. and Walt et al., ranges from 0.3 to 1.2% [28, 30]. A rare complication, such as bilhemia, occurs in less than 1% of patients with hepatic trauma [31]. Bilhemia is determined by the presence of pseudoaneurysm (that is found in angio CT), increased bilirubin level, and a normal level of liver enzymes (ALT, AST). We found the same frequency of bilhemia and hemobilia. Subcapsular hematoma should be surgically treated only for two reasons: (1) to increase the value of ALT and AST and (2) if the patient has Budd-Chiari syndrome [32, 33]. In our study, only one patient underwent surgical intervention due to increases in ALT and AST values.

The incidence of failure of conservative treatment due to associated intra-abdominal injuries has been reported to be between 0.5 and 3.5% [11, 34], whereas in our study, the failure rate with associated intra-abdominal injuries was 2.3%.

## Conclusions

The likelihood of the success of conservative treatment has a significant correlation with the grade of liver injury and associated intra-abdominal injuries. Limited hospital resources and the low level of consensus for non-surgical management have a negative impact on the outcome. The main causes of failure are secondary hemorrhage and bile leakage.
